# Cryptic *Plutella* species show deep divergence despite the capacity to hybridize

**DOI:** 10.1186/s12862-018-1183-4

**Published:** 2018-05-29

**Authors:** Kym D. Perry, Gregory J. Baker, Kevin J. Powis, Joanne K. Kent, Christopher M. Ward, Simon W. Baxter

**Affiliations:** 1 School of Agriculture, Food and Wine, The University of Adelaide, Adelaide, 5005 Australia; 2 School of Biological Sciences, The University of Adelaide, Adelaide, 5005 Australia; 3 South Australian Research and Development Institute, Adelaide, 5001 Australia

**Keywords:** *Plutella australiana*, *Plutella xylostella*, Lepidoptera, hybridization, sympatric, insecticide resistance, *Wolbachia*

## Abstract

**Background:**

Understanding genomic and phenotypic diversity among cryptic pest taxa has important implications for the management of pests and diseases. The diamondback moth, *Plutella xylostella* L., has been intensively studied due to its ability to evolve insecticide resistance and status as the world’s most destructive pest of brassicaceous crops. The surprise discovery of a cryptic species endemic to Australia, *Plutella australiana* Landry & Hebert, raised questions regarding the distribution, ecological traits and pest status of the two species, the capacity for gene flow and whether specific management was required. Here, we collected *Plutella* from wild and cultivated brassicaceous plants from 75 locations throughout Australia and screened 1447 individuals to identify mtDNA lineages and *Wolbachia* infections. We genotyped genome-wide SNP markers using RADseq in coexisting populations of each species. In addition, we assessed reproductive compatibility in crossing experiments and insecticide susceptibility phenotypes using bioassays.

**Results:**

The two *Plutella* species coexisted on wild brassicas and canola crops, but only 10% of *Plutella* individuals were *P. australiana*. This species was not found on commercial *Brassica* vegetable crops, which are routinely sprayed with insecticides. Bioassays found that *P. australiana* was 19-306 fold more susceptible to four commonly-used insecticides than *P. xylostella*. Laboratory crosses revealed that reproductive isolation was incomplete but directionally asymmetric between the species. However, genome-wide nuclear SNPs revealed striking differences in genetic diversity and strong population structure between coexisting wild populations of each species. Nuclear diversity was 1.5-fold higher in *P. australiana*, yet both species showed limited variation in mtDNA. Infection with a single *Wolbachia* subgroup B strain was fixed in *P. australiana*, suggesting that a selective sweep contributed to low mtDNA diversity, while a subgroup A strain infected just 1.5% of *P. xylostella*.

**Conclusions:**

Despite sympatric distributions and the capacity to hybridize, strong genomic and phenotypic divergence exists between these *Plutella* species that is consistent with contrasting colonization histories and reproductive isolation after secondary contact. Although *P. australiana* is a potential pest of brassicaceous crops, it is of secondary importance to *P. xylostella*.

**Electronic supplementary material:**

The online version of this article (10.1186/s12862-018-1183-4) contains supplementary material, which is available to authorized users.

## Background

Cryptic species can show remarkable diversity in aspects of their ecology, behaviour, and at the level of the genome. They exist across metazoan taxa [1], including globally important arthropod pest taxa, such as whiteflies [2], disease-vectoring mosquitoes [3], fruit flies [4], thrips [5, 6] and mites [7, 8], some of which are characterised by cryptic species complexes. Discovering cryptic diversity has important consequences for estimates of global biodiversity, conservation planning, and the management of pests and diseases. Morphologically similar species can vary in pest status due to differences in genotypic and/or phenotypic traits that influence their host range and specificity, geographic distribution, the ability to vector diseases, or insecticide resistance [8–10]. Therefore, recognising cryptic species and the differences in their biology and ecology are essential for effective management, with important implications for public health, agriculture and trade.

The diamondback moth, *Plutella xylostella*, is the major pest of brassicaceous crops worldwide, costing an estimated US$4 to US$5 billion annually in direct losses and management costs [11, 12]. Insecticide resistance is widespread in *P. xylostella* populations around the world, fuelling wide-ranging research to develop alternative management tactics [11, 13]. *Plutella xylostella* was initially recorded in Australia in the late 1800s and rapidly became a widespread pest of *Brassica* vegetables, and then canola following its expanded production from the 1990s [14, 15]. Recently, Landry and Hebert [16], through mtDNA barcoding, identified a cryptic lineage of *Plutella* in Australia not detected in previous molecular studies of *P. xylostella* [14, 17–21]. Although external morphology was indistinguishable from *P. xylostella*, deep mtDNA divergence (8.6%), differences in genital morphology and endemism in Australia led them to describe a new species, *Plutella australiana* Landry & Hebert. *Plutella australiana* was originally collected together with *P. xylostella* in light trap samples in eastern Australia, suggesting at least some ecological overlap [16], but its biology, ecology and pest status were unknown.

The management of *P. xylostella* in Australian *Brassica* crops has been a significant challenge for decades [15, 22], but the discovery of *P. australiana* has made the relative abundance and pest status of both species in these crops uncertain. With rare exception, *P. xylostella* and allied species feed on plants in the order Brassicales, mainly within the family Brassicaceae [16, 23, 24], implying that the host range of *P. australiana* may include cultivated brassicas. Widespread resistance to pyrethroid and organophosphate insecticides has been attributed to Australian populations of *P. xylostella* from all vegetable and canola production regions, which has led to ineffective control during outbreaks [22, 25]. *Plutella xylostella* is well known as a migratory insect with a high capacity for gene flow [11, 13], facilitating the rapid spread of resistance alleles. Australian *P. xylostella* are thought to disperse frequently, based on indirect evidence from ecological and genetic studies [14, 15, 26]. Most studies have found a lack of genetic differentiation at microsatellite loci and low sequence variation in mitochondrial DNA markers among Australian and New Zealand populations of *P. xylostella*, consistent with high gene flow and/or recent ancestry [14, 15, 17, 18]. While species identification was not in question in these studies, somewhat inconsistent findings in two studies from eastern Australia using allozymes or SSR markers [19, 20] might reflect the confounding presence of *P. australiana* samples [16]. Given these considerations, future management of *Plutella* in Australian crops will require thorough understanding of the ecological requirements, genetic traits and pest status of the two *Plutella* species. In addition, reproductive isolation between these two species is unknown but has implications for evolutionary inference and the potential for gene flow. The capacity for hybridization and introgression could lead to the exchange of insecticide resistance or other adaptive alleles [27, 28].

Although mtDNA markers are widely used in studies of species identity and population structure [29–31], mitochondrial variation within or between species can be influenced by direct and/or indirect selection, or introgressive hybridization [32, 33]. One factor that can confound mtDNA-based inference is interaction with inherited bacterial symbionts [34, 35]. *Wolbachia* is a widespread endosymbiont thought to infect at least half of arthropod [36] and 80% of lepidopteran [37] species. It is mainly transmitted vertically from infected females to their offspring through the egg cytoplasm, and inheritance is therefore linked with mtDNA. To facilitate its spread, *Wolbachia* manipulates host reproductive biology to favour the fitness of infected females by inducing host phenotypes that distort sex ratios (male-feminization, male-killing or induction of parthenogenesis) or cause sperm-egg cytoplasmic incompatibility (CI) [38, 39]. In the simple case involving a single CI-inducing strain, crosses with infected females are fertile but crosses between uninfected females and infected males fail to produce offspring. If maternal transmission is efficient and infected females have a reproductive advantage, *Wolbachia* infection can spread rapidly through an insect population [40], driving a selective sweep of a single haplotype and reducing mtDNA diversity [41]. Limited surveys to date have identified *Wolbachia* strains infecting *P. xylostella* at low frequency in populations from North America, Africa, Asia and Europe [18, 42, 43]. Because symbionts can contribute to reproductive isolation and influence mtDNA diversity [34, 44], assessing their role can provide important insights into host evolution and population structure [35, 45–47].

Here we investigated the biology, ecology and population genetic structure of two cryptic *Plutella* species by collecting *Plutella* from brassicaceous plants throughout Australia and screening individuals to identify mtDNA lineages and *Wolbachia* infections. For a subset of populations, we examined genetic diversity using thousands of nuclear SNPs from across the genome. In addition, we assessed reproductive compatibility in laboratory crosses and determined the susceptibility of each species to commercial insecticides.

## Methods

### Sample collection

*Plutella* larvae (rarely, eggs or pupae) were collected from canola crops, *Brassica* vegetable crops, forage brassicas and wild brassicas throughout Australia between March 2014 and December 2015 (Table 1). The wild species included wild radish, *Raphanus raphanistrum*, turnip weed, *Rapistrum rugosum*, sea rocket, *Cakile maritima*, Ward’s weed, *Carrichtera annua*, African mustard, *Brassica tournefortii*, and mixed stands of sand rocket, *Diplotaxis tenuifolia*, and wall rocket, *D. muralis*. At each location, at least 25 individuals were collected from randomly selected plants to achieve a representative sample. Insect samples were collected from *Brassica* vegetables by hand, from sea rocket by beating plants over a collection tray and from other hosts using a sweep net. Each population sample was separately reared in ventilated plastic containers on leaves of the original host material for 1–2 days and thereafter on cabbage leaves. Non-parasitised pupae or late-instar larvae were fresh frozen at −80 °C.

**Table 1:** Collection details showing the frequency (*f*) of *Plutella* species and *Wolbachia* infections among *Plutella* populations from Australia

						*P. australiana* ^b^	*P. xylostella*	
Location^a^	Collection date	Latitude	Longitude	Host	No. genotyped	No. (*f*)	No. (*f*)	No. (*f*) *wol*-infected
Boomi NSW	Sep-2014	-28.76°	149.81°	Canola	25	15 (0.60)	10 (0.40)	0 (0.00)
Gilgandra NSW	Sep-2014	-31.67°	148.72°	Wild turnip	23	21 (0.91)	2 (0.09)	0 (0.00)
Ginninderra NSW	Sep-2014	-35.19°	149.05°	Canola	15	2 (0.13)	13 (0.87)	0 (0.00)
Ginninderra NSW	Oct-2015	-35.19°	149.05°	Canola	34	27 (0.79)	7 (0.21)	0 (0.00)
Goulburn NSW	Nov-2015	-34.84°	149.67°	Canola	32	25 (0.78)	7 (0.22)	0 (0.00)
Henty NSW	Oct-2014	-35.60°	146.95°	Canola	18	1 (0.06)	17 (0.94)	0 (0.00)
Narromine NSW	Sep-2014	-32.22°	148.03°	Canola	26	0 (0.00)	26 (1.00)	1 (0.04)
Richmond NSW	Oct-2015	-33.60°	150.71°	Cabbage	21	0 (0.00)	21 (1.00)	0 (0.00)
Wagga Wagga NSW	Sep-2014	-35.04°	147.33°	Canola	21	5 (0.24)	16 (0.76)	0 (0.00)
Werombi NSW	Nov-2014	-33.99°	150.64°	Vegetables	16	0 (0.00)	16 (1.00)	0 (0.00)
Werombi NSW	Oct-2015	-34.00°	150.56°	Kale	13	4 (0.31)	9 (0.69)	0 (0.00)
Bundaberg QLD	Oct-2014	-24.80°	152.26°	Canola	14	1 (0.07)	13 (0.93)	0 (0.00)
Bundaberg QLD	Sep-2015	-24.80°	152.26°	Canola	30	0 (0.00)	30 (1.00)	0 (0.00)
Cunnamulla QLD	Sep-2015	-28.07°	145.68°	African mustard	17	17 (1.00)	0 (0.00)	0 –
Dalby QLD	Sep-2014	-27.28°	151.13°	Canola	30	0 (0.00)	30 (1.00)	0 (0.00)
Gatton QLD	Oct-2014	-27.54°	152.33°	Broccoli	16	0 (0.00)	16 (1.00)	0 (0.00)
Gatton QLD	Nov-2015	-27.54°	152.33°	Broccoli	15	0 (0.00)	15 (1.00)	0 (0.00)
Warwick QLD	Oct-2015	-28.21°	152.11°	Canola	16	0 (0.00)	16 (1.00)	0 (0.00)
Calca SA	Apr-2014	-33.02°	134.28°	Sand rocket, Wall rocket	13	8 (0.62)	5 (0.38)	0 (0.00)
Cocata SA	Sep-2014	-33.20°	135.13°	Canola	18	0 (0.00)	18 (1.00)	0 (0.00)
Colebatch SA	Feb-2015	-35.97°	139.66°	Forage brassica	18	0 (0.00)	18 (1.00)	0 (0.00)
Coonalpyn SA	Oct-2015	-35.62°	139.91°	Wild radish	11	0 (0.00)	11 (1.00)	0 (0.00)
Cowell SA	Sep-2014	-33.66°	137.16°	Canola	16	0 (0.00)	16 (1.00)	0 (0.00)
Keith SA	Oct-2014	-36.09°	140.29°	Canola	32	0 (0.00)	32 (1.00)	6 (0.19)
Lameroo SA	Sep-2014	-35.32°	140.51°	Canola	16	0 (0.00)	16 (1.00)	0 (0.00)
Lameroo SA	Oct-2015	-35.17°	140.48°	Canola	14	0 (0.00)	14 (1.00)	0 (0.00)
Littlehampton SA	Oct-2014	-35.06°	138.90°	Cabbage	34	0 (0.00)	34 (1.00)	6 (0.18)
Littlehampton SA	Sep-2015	-35.06°	138.90°	Brussels sprouts	8	0 (0.00)	8 (1.00)	0 (0.00)
Loxton SA	Sep-2014	-34.37°	140.72°	Canola	31	0 (0.00)	31 (1.00)	0 (0.00)
Loxton SA	Oct-2015	-34.50°	140.80°	Canola	14	1 (0.07)	13 (0.93)	0 (0.00)
Mallala SA	Sep-2015	-34.38°	138.50°	Canola	26	0 (0.00)	26 (1.00)	0 (0.00)
Meribah SA	Sep-2014	-34.74°	140.82°	Canola	16	0 (0.00)	16 (1.00)	0 (0.00)
Millicent SA	Apr-2015	-37.61°	140.34°	Canola	9	0 (0.00)	9 (1.00)	2 (0.22)
Minnipa SA	Oct-2015	-32.81°	135.16°	Canola	22	1 (0.05)	21 (0.95)	0 (0.00)
Moonaree SA	Aug-2014	-31.99°	135.87°	Ward’s weed	16	0 (0.00)	16 (1.00)	0 (0.00)
Mt Hope SA	Sep-2014	-34.14°	135.33°	Canola	29	0 (0.00)	29 (1.00)	0 (0.00)
Mt Hope SA	Sep-2015	-34.20°	135.34°	Canola	16	0 (0.00)	16 (1.00)	0 (0.00)
Padthaway SA	Oct-2015	-36.56°	140.43°	Canola	18	2 (0.11)	16 (0.89)	0 (0.00)
Picnic Beach SA	Apr-2014	-34.17°	135.27°	Sea rocket	2	0 (0.00)	2 (1.00)	0 (0.00)
Picnic Beach SA	Sep-2014	-34.17°	135.27°	Sea rocket	16	0 (0.00)	16 (1.00)	0 (0.00)
Redbanks SA	Oct-2014	-34.49°	138.59°	Canola	38	0 (0.00)	38 (1.00)	1 (0.03)
Sherwood SA	Oct-2014	-36.05°	140.64°	Wild radish	8	0 (0.00)	8 (1.00)	0 (0.00)
Southend SA	Apr-2015	-37.57°	140.12°	Sea rocket	18	0 (0.00)	18 (1.00)	0 (0.00)
Tintinara SA	Oct-2015	-35.97°	139.66°	Forage *Brassica*	17	0 (0.00)	17 (1.00)	0 (0.00)
Ucontichie SA	Sep-2014	-33.22°	135.31°	Canola	3	0 (0.00)	3 (1.00)	0 (0.00)
Virginia SA	Oct-2014	-34.64°	138.54°	Broccoli	18	0 (0.00)	18 (1.00)	1 (0.06)
Virginia SA	Sep-2015	-34.64°	138.54°	Cabbage	23	0 (0.00)	23 (1.00)	0 (0.00)
Walkers Beach SA	Sep-2014	-33.55°	134.86°	Sea rocket	16	0 (0.00)	16 (1.00)	0 (0.00)
Walkers Beach SA	Mar-2015	-33.55°	134.86°	Sea rocket	16	0 (0.00)	16 (1.00)	0 (0.00)
Walkers Beach SA	Sep-2015	-33.55°	134.86°	Sea rocket	19	0 (0.00)	19 (1.00)	0 (0.00)
Wirrabara SA	Oct-2014	-32.99°	138.31°	Canola	28	2 (0.07)	26 (0.93)	0 (0.00)
Wokurna SA	Sep-2015	-33.67°	137.96°	Wild radish	24	1 (0.04)	23 (0.96)	0 (0.00)
Wurramunda SA	Apr-2014	-34.30°	135.56°	Wild canola	16	0 (0.00)	16 (1.00)	0 (0.00)
Deddington TAS	Nov-2014	-41.59°	147.44°	Kale	16	0 (0.00)	16 (1.00)	0 (0.00)
Launceston TAS	Nov-2014	-41.47°	147.14°	Wild mustard	16	0 (0.00)	16 (1.00)	0 (0.00)
Newstead TAS	Nov-2015	-41.59°	147.44°	Cauliflower	22	0 (0.00)	22 (1.00)	0 (0.00)
Cowangie VIC	Oct-2015	-35.10°	141.33°	Canola	19	0 (0.00)	19 (1.00)	0 (0.00)
Ouyen VIC	Sep-2014	-35.00°	142.31°	Canola	28	1 (0.04)	27 (0.96)	0 (0.00)
Robinvale VIC	Sep-2014	-34.81°	142.94°	Canola	16	0 (0.00)	16 (1.00)	0 (0.00)
Werribee VIC	Oct-2014	-37.94°	144.73°	Cauliflower	16	0 (0.00)	16 (1.00)	0 (0.00)
Werribee VIC	Nov-2015	-37.94°	144.73°	Cauliflower	16	0 (0.00)	16 (1.00)	0 (0.00)
Yanac VIC	Sep-2014	-36.06°	141.25°	Canola	17	0 (0.00)	17 (1.00)	0 (0.00)
Boyup Brook WA	Sep-2014	-33.64°	116.40°	Canola	26	2 (0.08)	24 (0.92)	0 (0.00)
Dalwallinu WA	Sep-2015	-30.28°	116.66°	Canola	20	0 (0.00)	20 (1.00)	0 (0.00)
Dalyup WA	Oct-2015	-33.72°	121.64°	Wild radish	22	3 (0.14)	19 (0.86)	0 (0.00)
Esperance WA	Sep-2014	-33.29°	121.76°	Canola	23	8 (0.35)	15 (0.65)	1 (0.07)
Esperance WA	Oct-2015	-33.79°	122.13°	Canola	16	0 (0.00)	16 (1.00)	0 (0.00)
Gingin WA	Dec-2014	-31.28°	115.65°	Red cabbage	23	0 (0.00)	23 (1.00)	1 (0.04)
Kalannie WA	Sep-2015	-30.00°	117.25°	Canola	18	0 (0.00)	18 (1.00)	0 (0.00)
Manjimup WA	Dec-2014	-34.18°	116.23°	Chinese cabbage	17	0 (0.00)	17 (1.00)	0 (0.00)
Manjimup WA	Nov-2015	-34.18°	116.23°	*Brassica* vegetables	13	0 (0.00)	13 (1.00)	0 (0.00)
Narrogin WA	Oct-2015	-32.95°	117.32°	Wild radish, wild canola	15	0 (0.00)	15 (1.00)	0 (0.00)
Narrogin WA	Oct-2015	-32.96°	117.33°	Canola	32	0 (0.00)	32 (1.00)	0 (0.00)
Walkaway WA	Sep-2014	-28.94°	114.83°	Canola	19	0 (0.00)	19 (1.00)	0 (0.00)
Walkaway WA	Sep-2014	-28.16°	114.63°	Canola	16	0 (0.00)	16 (1.00)	0 (0.00)
Total					1447	147 (0.10)	1300 (0.90)	19 (0.01)

^a^Australian states: NSW = New South Wales, QLD = Queensland, SA = South Australia, TAS = Tasmania, VIC = Victoria, WA = Western Australia

^b^All *P. australiana* individuals were infected with *Wolbachia*

### DNA isolation and COI genotyping

For each population sample, we aimed to genotype a minimum of 16 individuals where possible after removing parasitized individuals. Individual pupae (but not larvae) were sexed under a dissecting microscope, then genomic DNA was isolated by homogenising whole individuals followed by two phenol and one chloroform extractions according to Zraket et al. [48]. DNA was treated with RNase A, then precipitated and re-suspended in TE buffer. *Plutella* lineages were distinguished using a PCR-RFLP assay [49]. A 707 bp COI region was amplified using a combination of two primer pairs: (i) PxCOIF (5^′^-TCAACAAATCATAAAGATATTGG- 3^′^) and PxCOIR (5^′^-TAAACTTCAGGGTGACCAAAAAATCA- 3^′^), and (ii) PaCOIF (5^′^-TCAACAAATCATAAGGATATTGG- 3^′^) and PaCOIR (5 ^′^-TAAACCTCTGGATGGCCAAAAAATCA- 3^′^). Ten microliter reactions were run with 2 µL of MyTaq 5x buffer, 0.2 µL of each primer (10mM stocks), 1 µL of DNA (approx. 5 ng) and 0.05 µL of MyTaq polymerase (Bioline). Samples were amplified at 95 °C for 2 min, then 35 cycles at 95 °C for 10 s, 52 °C for 20 s, 72 °C for 30 s followed by a 5 min final extension at 72 °C. PCR products were digested at 37 °C for 1 h with 1 unit of *AccI* (NEB) restriction enzyme with 2 µL Cutsmart Buffer in a 20 µL reaction. Following digestion, products were separated using agarose gel electrophoresis (1.5%). *Plutella xylostella* products are approximately 516 bp and 191 bp and *P. australiana* products are 348 bp and 359 bp [49]. To examine mtDNA haplotypes, sequencing of the 707 bp COI amplicon was performed for 44 *P. xylostella* and 37 *P. australiana* individuals at the Australian Genome Research Facility (AGRF). In addition, we downloaded sequence trace files from Landry and Hebert [16] (dx.doi.org/10.5883/DS-PLUT1) and re-analysed, aligned and trimmed all sequences in GENEIOUS version 10.0.6 [50]. Haplotype networks were constructed using R package pegas version 0.9 [51].

### *Wolbachia* screening and phylogenetics

*Wolbachia* infection was detected using two separate PCR assays of the 16S rRNA gene (16S-2 and 16S-6) according to Simoes et al. [52]. To identify *Wolbachia* strains, the *Wolbachia surface protein* (*wsp*) gene was sequenced in a subset of individuals. Amplification was performed using wsp81F and wsp691R sequence primers [53]. Amplicons were sequenced using the reverse primer and aligned in GENEIOUS version 10.0.6 [50]. We used a 493 bp alignment to construct a maximum likelihood phylogeny in RAxML version 8.2.4 [54] using a general time reversal substitution model [55] with 1000 bootstrap replicates.

### RADseq library preparation and sequencing

Libraries were prepared for restriction-site-associated DNA sequencing (RADseq) according to a protocol modified from Baird et al. [56]. Genomic DNA was quantified using a Qubit 2.0 fluorometer (Invitrogen) and 200 ng digested with 10 units of high fidelity *SbfI* in Cutsmart Buffer (NEB) for 1 h at 37 °C, then heat inactivated at 80 °C for 20 min. One microlitre of P1 adapter (100nM) with a 6-base molecular identifier (MID) (top strand 5^′^-TCGTCGGCAGCGTCAGATGTGTATAAGAGACAGxxxxxxTGCA- 3^′^, bottom strand 5^′^-[P]xxxxxxCTGTCTCTTATACACATCTGACGCTGCCGACGA- 3^′^, x represents sites for MIDs) were then added using 0.5 µL T4 DNA ligase (Promega), 1 nM ATP and Cutsmart buffer. Library pools were sheared using a Bioruptor sonicator (Diagenode), then DNA fragments end-repaired using a Quick Blunting Kit (NEB), adenine overhangs added then P2 adapters (top strand 5^′^-[P]CTGTCTCTTATACACATCTCCAGAATAG- 3^′^, bottom strand 5^′^-GTCTCGTGGGCTCGGAGATGTGTATAAGAGACAGT- 3^′^) ligated. DNA purification between steps was performed using a MinElute PCR purification kit (Qiagen). Libraries were amplified using KAPA HiFi Hotstart Readymix (Kapa Biosystems) and Nextera i7 and i5 indexed primers with PCR conditions: 95 °C for 3 min, two cycles of 98 °C for 20 s, 54 °C for 15 s, 72 °C for 1 min, then 15 cycles of 98 °C for 20 s, 65 °C for 15 s, 72 °C for 1 min followed by a final extension of 72 °C for 5 min. Libraries were size-selected (300-700 bp) on 1–1.5% agarose gel and purified using a minElute Gel Extraction Kit (Qiagen), then Illumina paired-end sequencing was performed using HiSeq2500 (100 bp) or NextSeq500 (75 bp) at the AGRF.

### Read filtering and variant calling

Sequence reads were demultiplexed using RADtools version 1.2.4 [57] allowing one base MID mismatch, then TRIMMOMATIC version 0.32 [58] was used to remove restriction sites, adapter sequences and a thymine base from reverse reads introduced by the P2 adapter, and quality filter using the ILLUMINACLIP tool with parameters: TRAILING:10 SLIDINGWINDOW:4:15 MINLEN:40. Paired reads were aligned to the *P. xylostella* reference genome (accession number: GCF_000330985.1) using STAMPY version 1.0.21 [59] with --baq and --gatkcigarworkaround options and expected substitution rate set to 0.03 for *P. xylostella* and 0.05 for *P. australiana* to reflect expected levels of sequence divergence relative to the *P. xylostella* reference genome. Duplicate reads were removed using PICARD version 1.71 [60]. Genotypes were called using the Genome Analysis Toolkit (GATK) version 3.3-0 [61, 62] HaplotypeCaller tool. We determined that base quality score recalibration using bootstrapped SNP databases was inappropriate for this dataset as it globally reduced quality scores. For downstream comparisons between species, we joint-genotyped *P. australiana* and *P. xylostella* individuals using the GATK GenotypeGVCFs workflow. To examine finer scale population structure, we also joint-genotyped the *P. australiana* individuals alone. All variant callsets were hard-filtered with identical parameters using VCFtools version 0.1.12a [63]: We removed indels and retained confidently-called biallelic SNPs (GQ$\geqslant $30) genotyped in at least 70% of individuals with a minimum genotype depth of 5, minQ$\geqslant $400, average site depth of 12–100, minimum minor allele frequency of 0.05, in Hardy-Weinberg equilibrium at an alpha level of 0.05. To avoid linked sites, we used the VCFtools --thin function to retain only SNPs separated by a minimum of 2000 bp. To estimate genetic diversity, we generated a set of all confidently-called variant and invariant sites (GQ$\geqslant $30), and hard filtered to remove sites within repetitive regions and retain sites genotyped in at least 70% of individuals with an average site depth of 12–100. Sites from the mitochondrial genome were excluded from all datasets.

### Genetic diversity and population structure

Genetic diversity was calculated for *Plutella* populations of both species from five locations. The R package hierfstat [64] was used to calculate observed heterozygosity, gene diversity and the inbreeding coefficient, *F*_IS_, according to Nei [65]. Population means for site depth and number of SNPs, indels and private sites were calculated using the --depth function and vcfstats module in VCFtools version 0.1.12a [63]. The number of heterozygous sites within individuals was determined from all confidently-called sites excluding indels using a custom python script parseVCF.py [66] and visualised using R [67].

To examine population structure in *P. australiana*, a global estimate of *F*_ST_ [68] with bootstrapped 99% confidence intervals (10^4^ bootstrap replicates) was calculated in R package diveRsity [69]. Pairwise *F*_ST_ values for all population pairs were calculated and significance determined using exact *G* tests (10^4^ mc burnins, 10^3^ batches, and 10^4^ iterations per batch) in GENEPOP version 4.6 [70] after Bonferroni correction for multiple comparisons. Separate analysis of population structure was performed using the Bayesian clustering program STRUCTURE version 2.3.4 [71], first for all individuals of co-occurring *Plutella* species, and second for *P. australiana* alone. For all runs, we used a burnin length of 5×10^5^ followed by a run length of 10^6^ MCMC iterations and performed ten independent runs for each *K* value from 1 to 10, where *K* is the number of genotypic clusters, using a different random seed for each run, assuming the locprior model with correlated allele frequencies and *λ* set to 1. The optimal value of *K* was determined using the delta *K* method [72] implemented in STRUCTURE HARVESTER [73] and inspection of the likelihood distribution for each model. *Q*-matrices were aligned across runs using CLUMPP version 1.1.2 [74] and visualised using DISTRUCT version 1.1 [75].

### Laboratory cultures of *Plutella* species

Laboratory cultures of *P. australiana* and *P. xylostella* were established from field populations and used for crossing experiments and insecticide bioassays. *Plutella* adults were collected at light traps at Angle Vale and Urrbrae, South Australia, in October–November 2015. Females were isolated and allowed to lay eggs, then identified using PCR-RFLP and progeny pooled to produce separate cultures of each species. A laboratory culture of the Waite Susceptible *P. xylostella* strain (S) has been maintained on cabbage without insecticide exposure for approximately 24 years (≈310 generations) and was used as a bioassay reference strain. All cultures were maintained in laboratory cages at 26 ± 2.0°C and a 14:10 (L:D) hour photoperiod at the South Australian Research and Development Institute, Waite Campus, Adelaide, South Australia. The *P. australiana* culture was maintained on sand rocket, *Diplotaxis tenuifolia*, and the *P. xylostella* culture was maintained on cabbage, *Brassica oleracea* var. *capitata*. The purity of cultures was assessed regularly using PCR-RFLP.

### Crossing experiments

*Plutella australiana* and *P. xylostella* pupae were sexed under a stereo microscope, then placed into individual 5 mL clear polystyrene tubes with fine mesh lids and gender visually confirmed after eclosion. Enclosures used for crossing experiments were 850 mL polypropylene pots (Bonson Pty Ltd) modified with lateral holes covered with voile mesh for ventilation. Crosses of single mating pairs were performed on laboratory benches at 26 ± 2.0 °C and 14:10 (L:D) photoperiod using 3-week old *D. tenuifolia* seedlings as the host plant. After seven days, adults were collected into a 1.5 mL tube and fresh frozen at −80 °C for species confirmation using PCR-RFLP. Seedlings were examined and eggs counted under a stereo microscope, then returned to enclosures to allow egg hatch. Larvae were provided with fresh 3–4 week old seedlings until pupation, then pupae were individually collected into 5 mL tubes. Hybrid F1xF1 crosses and back-crosses were then performed as above. The presence of egg and adult offspring was recorded for all replicates, and for the majority of replicates (> 80*%*), the numbers of offspring were counted and used to calculate a mean.

### Insecticide bioassays

Insecticide susceptibility of field-collected *Plutella* strains was compared to the susceptible *P. xylostella* (S) reference in dose-response assays using four commercial insecticides: Dominex (100 g L^−1^ alpha-cypermethrin), Proclaim (44 g kg^−1^ emamectin benzoate), Coragen (200 g L^−1^ chlorantraniliprole) and Success Neo (120 g L^−1^ spinetoram). Bioassays were performed by placing 3^*rd*^ instar larvae onto inverted leaf discs embedded in 1% agar in 90 mm Petri dishes. Cabbage leaves, *Brassica oleracea*. var. *capitata* were used for *P. xylostella* and canola leaves, *B. napus* var. ‘ATR Stingray’, were used for *P. australiana*. Eight concentrations and a water-only control were evaluated for each insecticide using four replicates of ten larvae. A 4 mL aliquot of test solution was applied directly to leaves using a Potter Spray Tower (Burkard Manufacturing Co. Ltd.) calibrated to deliver an aliquot of 3.52 ± 0.09 mg cm^-1^. After application, each dish was placed in a controlled temperature room at 25 ± 0.5 °C, then mortality was assessed after 48 h (Dominex, Success Neo) or 72 h (Proclaim, Coragen). Dose-response analysis was performed using log-logistic regression in R package drc [76] and the fitted models were used to estimate the lethal concentration predicted to cause 50% (*LC*_50_) and 99% (*LC*_99_) mortality of the test population. Resistance ratios were calculated by dividing the *LC*_50_ and *LC*_99_ estimates for field strains by the corresponding *LC* estimates for the *P. xylostella* (S) reference strain.

## Results

### Geographic distribution and host associations

**Fig. 1:**
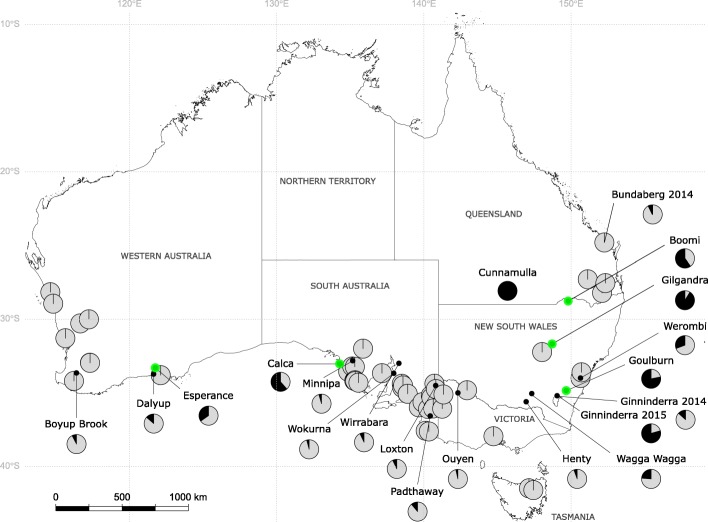
The geographic distribution of *P. xylostella* (light grey) and *P. australiana* individuals (black) in larval collections from brassicaceous plants in Australia during 2014 and 2015. Pie diagrams show the relative proportion of each species at each location. Overlapped pies represent locations with 100% *P. xylostella*. Green highlighted circles indicate five locations from which individuals of each species were RAD sequenced

*Plutella* larvae were collected from brassicaceous plants at 75 locations in Australia and 1477 individuals were genotyped at the COI locus using PCR-RFLP to identify species. Of these, 88% (*n*=1300) were genotyped as *P. xylostella*, 10% (*n*=147) as *P. australiana* and 2% (*n*=30) were unresolved (Table 1). *Plutella australiana* was identified in around one quarter (*n*=20/75) of collections distributed across southern Australia, while *P. xylostella* occurred at all locations except Cunnamulla, Queensland, in a collection from wild African mustard, *Brassica tournefortii* (Table 1). The sex ratio was not different from 1:1 for *P. xylostella* (481 females, 517 males, *χ*^2^=1.2986, *p*=0.2545) or *P. australiana* (63 females, 55 males, *χ*^2^=0.5424, *p*=0.4615). The relative incidence and abundance of *P. australiana* was >2-fold higher in the eastern state of New South Wales than in other states (Fig. 1). *Plutella australiana* larvae were detected in 29% (*n*=5/17) of collections from wild brassicas and from species including wild radish, *Raphanus raphanistrum*, wild turnip, *Rapistrum rugosum*, African mustard, *B. tournefortii*, and mixed stands of sand rocket, *D. tenuifolia* and wall rocket, *D. muralis* (Table 2). Among cultivated crops, *P. australiana* larvae occurred in 36% (*n*=14/39) of samples from canola, consisting of 11% of total *Plutella* individuals from those crops, but were not identified from commercial *Brassica* vegetable farms (Table 2). However, *P. australiana* eggs were collected from kale at one farm.

**Table 2:** Frequency of *P. australiana* in *Plutella* collections from different *Brassica* host types

Host	No.	No. *P.aus*	No.	No. *P.aus*
	locations	locations	genotyped	
Wild brassicas	17	5 (0.29)	268	50 (0.19)
Canola crops	39	14 (0.36)	848	93 (0.11)
Vegetable crops	16	1 (0.06)	287	4 (0.01)
Forage brassicas	3	0 (0.00)	44	0 (0.00)

Presented are the numbers and proportion in parentheses of *P. australiana* across collection locations and individuals genotyped

### *Wolbachia* infections

**Fig. 2:**
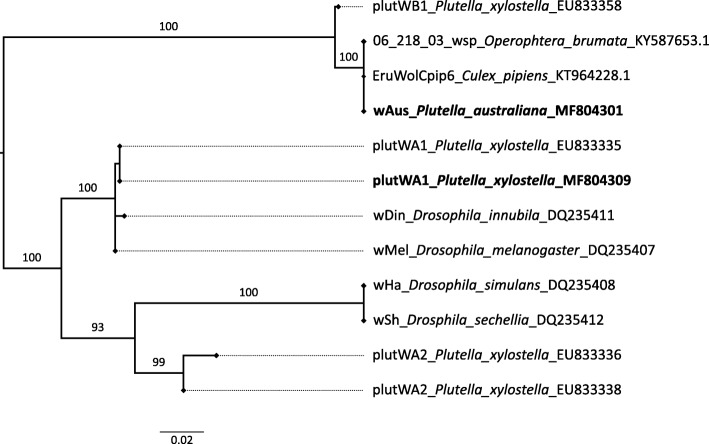
Maximum likelihood phylogeny of 493 bp of *Wolbachiawsp* amplicons for *Plutella* and other arthropods. The strain infecting *P. australiana* (*wAus*) was identical to a *Wolbachia* supergroup B strain reported from *Culex pipiens* and *Operophtera brumata*. The strain infecting Australian *P. xylostella* was identical to a supergroup A strain (*plutWA1*) reported from Malaysian *P. xylostella*. Labels include the *Wolbachia* strain, host species and GenBank accession number. Labels in bold denote strains sequenced in this study. The scale bar shows the mean number of nucleotide substitutions per site

*Plutella* individuals (*n*=1447) were screened for *Wolbachia* infection using 16S rRNA PCR assays. Only 1.5% (*n*=19/1300) of *P. xylostella* collected from eight different locations were infected (Table 1). In contrast, all 147 *P. australiana* individuals were infected with *Wolbachia* across the 20 locations where this species occurred. To identify *Wolbachia* strains, a *Wolbachia surface protein* (*wsp*) amplicon was sequenced from 14 *P. xylostella* and 30 *P. australiana* individuals. Each species was infected with a different strain. The *wsp* sequence for Australian *P. xylostella* showed 100% identity to a *Wolbachia* supergroup A isolate infecting *P. xylostella* from Malaysia, *plutWA1* [18]. For *P. australiana*, the *wsp* sequence showed 100% identity to a *Wolbachia* supergroup B isolate infecting a mosquito, *Culex pipiens*, from Turkey and the winter moth, *Operophtera brumata*, from the Netherlands (Fig. 2).

### Crossing experiments

Inter-species single pair mating experiments showed that hybridization between *P. australiana* and *P. xylostella* was possible, yet less successful than intra-species crosses. While most intra-species crosses produced adult offspring, the fecundity of *P. xylostella* was >2-fold higher than *P. australiana* (Table 3). Both reciprocal inter-species crosses produced F1 adult offspring, but success was asymmetric and notably higher in the pairs with *P. australiana* females. In this direction, there was a strong male bias in the F1 progeny: from 76 cross replicates, 16 collectively produced 9 female and 80 male adults, a ratio of 8.9. Hybrid F1xF1 crosses for both parental lines produced F2 adult offspring (Table 4). For the *P. australiana* maternal line, parental back-crosses using F1 hybrid males successfully produced offspring, while parental back-crosses with F1 hybrid females were sterile. For the *P. xylostella* maternal line, low fitness allowed only a single parental back-cross replicate, which involved a hybrid female and was sterile.

**Table 3:** Fecundity of intra-species and reciprocal inter-species single pair crosses of *P. australiana* (*P.aus*) and *P. xylostella* (*P.x*)

Cross (♀ × ♂)	No. replicates	No. reps eggs	No. reps adults	Mean ± SEM no. eggs	Mean ± SEM no. adults
*P.aus*♀ ×*P.aus*♂	42	37 (0.881)	34 (0.81)	40.86 ±5.33	9.66 ±1.7
*P.x*♀ ×*P.x*♂	63	59 (0.937)	59 (0.937)	83.82 ±10.61	24.28 ±3.27
*P.aus*♀ ×*P.x*♂	76	49 (0.645)	16 (0.211)	18.43 ±3.02	1.17 ±0.33
*P.x*♀ ×*P.aus*♂	85	62 (0.729)	3 (0.035)	15.16 ±2.37	0.06 ±0.03

Presented are the number and proportion in parentheses of replicates (reps) that produced eggs and adult offspring, and the mean ± standard error of the mean number of eggs and adult offspring per replicate

**Table 4:** Fecundity of hybrid F1 crosses and back-crosses

Cross (♀ × ♂)	No. replicates	No. reps eggs	No. reps adults	Mean ± SEM no. eggs	Mean ± SEM no. adults
F0 *P.aus*♀ source					
(*P.aus* ×*P.x*♂)♀ × (*P.aus* ×*P.x*♂)♂	4	4 (1.000)	2 (0.500)	66.00 ±60.00	–
(*P.aus* ×*P.x*♂)♀ ×*P.aus*♂	7	7 (1.000)	0 (0.000)	20.33 ±11.86	0.00 ±0.00
*P.aus*♀ × (*P.aus* ×*P.x*♂)♂	9	5 (0.556)	2 (0.222)	6.38 ±3.54	0.22 ±0.44
(*P.aus* ×*P.x*♂)♀ ×*P.x*♂	4	4 (1.000)	0 (0.000)	39.00 ±19.00	0.00 ±0.00
*P.x*♀ × (*P.aus* ×*P.x*♂)♂	15	15 (1.000)	4 (0.267)	36.75 ±3.21	0.33 ±0.62
F0 *P.x*♀ source					
(*P.x* ×*P.aus*♂)♀ × (*P.x* ×*P.aus*♂)♂	6	5 (0.833)	4 (0.667)	74.50 ±22.79	6.17 ±5.27
(*P.x* ×*P.aus*♂)♀ ×*P.aus*♂	1	0 (0.000)	0 (0.000)	0.00	0.00

Presented are the number and proportion in parentheses of replicates (reps) producing eggs and adult offspring, and the mean ± standard error of the mean numbers of eggs and adults offspring per replicate. A dash denotes an absence of count data

### Mitochondrial haplotype diversity

**Fig. 3:**
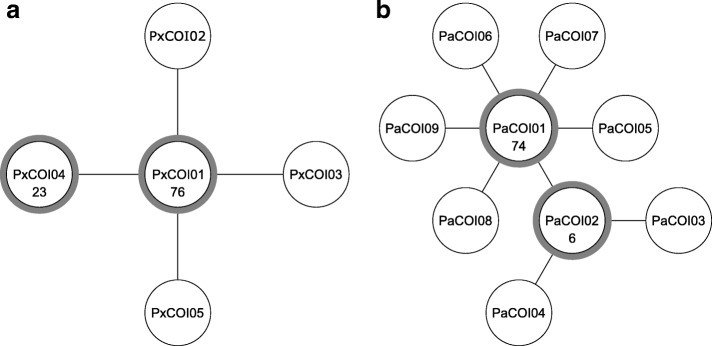
Mitochondrial DNA haplotype network for **a***P. xylostella* (n=102,44 from this study, 58 from [16]) and **b***P. australiana* (n=87, 37 from this study, 50 from [16]) individuals from Australia based on a 613 bp COI sequence alignment. Haplotypes shared by more than one individual are shown in circles with a grey border with the number of individuals indicated inside the circle. Haplotypes connected by a line differ by a single mutation

Mitochondrial haplotype networks of Australian *Plutella* were constructed using a 613 bp COI alignment that included 81 sequences from this study and 108 from Landry and Hebert [16]. We found low haplotype diversity within Australian *P. xylostella*, consistent with previous reports [17, 18, 77]. Only five haplotypes were identified among 102 individuals, including three identified by Saw et al. [17] and three occurring in single individuals (Fig. 3a). The most common haplotype, PxCOI01, occurred at high frequency and differed by a single base mutation from other haplotypes (Fig. 3a, Additional file 1: Table S1). Nine closely related haplotypes were identified in 87 *P. australiana* individuals with seven occurring in single individuals (Fig. 3b). The most common haplotype, PaCOI01, occurred at high frequency and differed by 1-2 base mutations from other haplotypes (Fig. 3b, Additional file 1: Table S2).

### Nuclear diversity and population structure

At five collection locations, *P. australiana* co-occurred with *P. xylostella* in sufficient numbers to enable comparison of nuclear genomes, though the relative abundance of species varied between locations. To ensure representation from the south-west region of Australia, the Esperance population (*n*=5) was formed by including one *P. australiana* individual from Boyup Brook. Despite only two *P. xylostella* individuals at Gilgandra, this population had 17 *P. australiana* individuals and was included. To generate nuclear SNP markers, we performed RADseq for a total of 52 *P. australiana* and 47 *P. xylostella* individuals.

Illumina sequencing and demultiplexing using RADtools [57] yielded 276.8 million raw sequence reads. Following read quality filtering and mapping, genotypes were called for 99 individuals from the two species. Hard filtering retained 300,241 confidently-called variant and invariant nuclear sites at a mean depth >36 per individual, and a subset of 689 widely-dispersed nuclear SNP variants (to avoid linkage bias) at a mean depth >36 per individual, for comparative analyses of genetic diversity and population structure. The dataset with all confidently-called sites was used to estimate population-level genetic diversity.

**Fig. 4:**
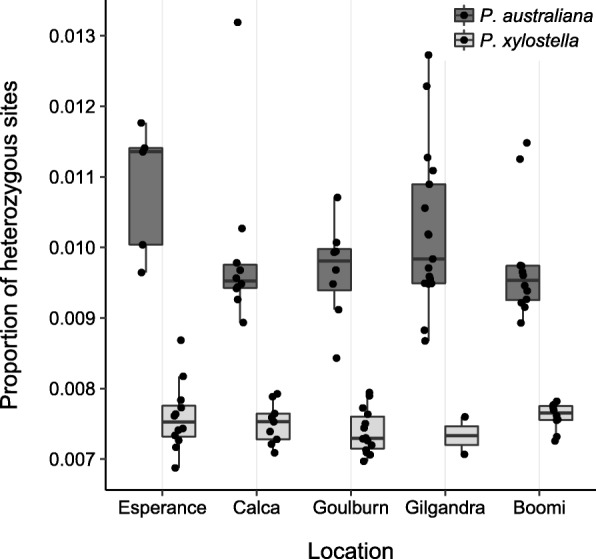
Boxplot showing the proportion of heterozygous sites across 289,347 confidently-called nuclear sites for individuals of *P. xylostella* (light grey boxes, n=47) and *P. australiana* (dark grey boxes, n=52) from five locations. Heterozygosity was consistently higher in *P. australiana*

Estimates of nuclear genetic diversity across 300,241 variant and invariant sites revealed a striking contrast between *Plutella* species, with notably higher diversity within populations of *P. australiana* than co-occurring populations of *P. xylostella* (Table 5). The mean observed heterozygosity within populations ranged from 0.013–0.016 for *P. australiana* and 0.009–0.010 for *P. xylostella*. Similarly, the average numbers of SNPs, indels and private alleles were considerably higher within *P. australiana* populations. As *P. australiana* may have fixed nucleotide differences relative to the *P. xylostella* reference genome that may affect population level statistics, we also removed indels from this dataset and directly compared the heterozygosity among individuals using 289,347 sites. *Plutella australiana* individuals had on average a 1.5-fold higher proportion of heterozygous sites than *P. xylostella* individuals (Fig. 4).

**Table 5:** Population statistics for variant and invariant sites for sympatric populations of *P. australiana* (*P. aus*) and *P. xylostella* (*P. x*) from five locations

Population	Species	*n*	Sites	Site depth	SNPs	Indels	Private sites	*H*_o_	*H*_s_	*F*_is_
Boomi NSW	*P.* *aus*	11.1	276939	40	7198	1112	212	0.013	0.015	0.089
	*P.* *x*	9.4	282989	42	4316	549	30	0.009	0.010	0.039
Calca SA	*P.* *aus*	8.7	261496	30	6629	989	210	0.014	0.015	0.059
	*P.* *x*	8.2	274973	42	4126	538	40	0.009	0.010	0.050
Esperance WA	*P.* *aus*	4.5	269268	28	6543	998	210	0.016	0.015	-0.032
	*P.* *x*	11.0	275299	35	4046	520	23	0.010	0.010	0.019
Gilgandra NSW	*P.* *aus*	15.7	277136	39	7154	1088	212	0.014	0.015	0.079
	*P.* *x*	1.9	277846	42	4149	505	28	0.009	0.009	-0.056
Goulburn NSW	*P.* *aus*	6.8	256343	29	6471	968	190	0.013	0.015	0.058
	*P.* *x*	12.8	274700	36	4052	513	26	0.009	0.010	0.052

*n*, number of individuals genotyped per locus; *H*_o_, observed heterozygosity; *H*_s_, gene diversity; *F*_is_, Nei’s inbreeding coefficient

**Fig. 5:**
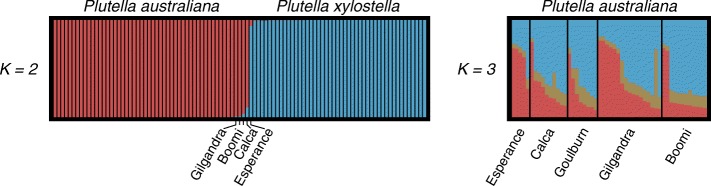
Proportional assignment of *Plutella* individuals to genotypic clusters, *K*, based on STRUCTURE analysis. Individuals are represented by vertical bars and genotypic clusters are represented by different colors. Left panel: Analysis at *K*=2 for 52 *P. australiana* and 47 *P. xylostella* individuals sorted left-to right by proportion of cluster membership. The predominantly red bars correspond to *P. australiana* individuals and the predominantly blue bars correspond to *P. xylostella* individuals identified through mtDNA genotypes. Locations are labelled for five individuals showing >1% genotypic admixture. Right panel: Analysis at *K*=3 for 52 *P. australiana* individuals sorted left-to-right by proportion of cluster membership within geographic locations, showing a high degree of genotypic admixture among individuals across locations

Genetic structure among co-occurring populations of *Plutella* species was investigated using 689 widely-dispersed nuclear SNPs in the program STRUCTURE. The delta *K* method predicted a strong optimal at *K*=2 genotypic clusters. *Plutella australiana* and *P. xylostella* individuals were clearly separated into distinct genotypic clusters in accordance to their species identified through mtDNA genotypes regardless of geographic location (Fig. 5, left panel). Five individuals across four locations showed greater than 1% admixture as shown by sharing of colored bars.

Assessing population structure from datasets with multiple species can mask heirachical structure [78]. To address this, genotypes were separately called for 52 *P. australiana* individuals, and hard filtering retained a set of 974 widely-dispersed SNP variants at a mean depth >33 per individual for examination of finer scale structure among five populations. The delta *K* method predicted a weak modal signal at *K*=3, but the highest likelihood value occurred at *K*=1. Bar plots for *K*=3 showed a high degree of admixture among individuals across the five populations, consistent with high levels of gene flow across Australia (Fig. 5, right panel). Pairwise *F*_ST_ was then calculated for the five *P. australiana* populations using 974 SNPs. The global estimate of *F*_ST_ was not significantly different from zero, indicating the populations are not differentiated (*F*_ST_=0.0002, 99% CI = -0.0274–0.0387). Further, pairwise *F*_ST_ values were low, ranging from –0.0041 to 0.0038, suggesting substantial gene flow among populations separated by distances of between 341 and 2700 kilometres (Table 6).

**Table 6:** Pairwise comparisons^a^ of Weir and Cockerham’s [68] *F*_ST_ (below diagonal) and geographic distance in kilometres (above diagonal) among populations of *P. australiana* from five locations

	Boomi	Calca	Esperance	Gilgandra	Goulburn
Boomi	–	1555	2714	341	677
Calca	-0.0041	–	1167	1365	1434
Esperance	0.0038	0.0014	–	2531	2572
Gilgandra	0.0000	0.0036	-0.0005	–	364
Goulburn	-0.0015	-0.0014	0.0034	0.0005	–

^a^Exact *G* tests were non-significant for all population pairs (*p*>0.05)

### Insecticide susceptibility

**Fig. 6:**
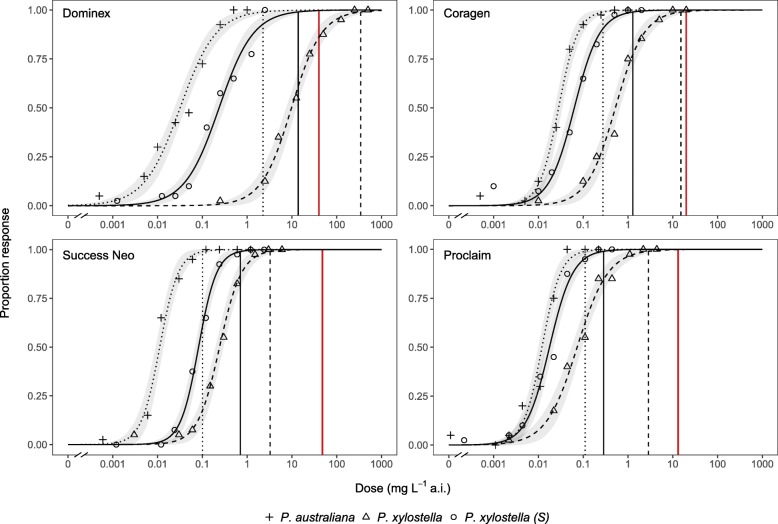
Insecticide bioassay dose-response curves for *P. australiana* (dotted line) and *P. xylostella* (dashed line) field strains collected from Angle Vale and Urrbrae, South Australia, and a susceptible *P. xylostella* (S) reference strain (solid line), exposed to four commercial insecticides: Dominex, Coragen, Proclaim and Success Neo. Points are the mean observed response across 4 bioassay replicates of 10 larvae each and lines are the fitted log-logistic response curves with 95% confidence intervals shown in grey shading. The vertical red line represents the approximate commercial field dose for each insecticide and vertical black lines represent the estimated *LC*_99_ for the corresponding *Plutella* strain

Bioassays revealed highly contrasting responses to insecticide exposure in *P. xylostella* and *P. australiana* field strains (Fig. 6). *Plutella australiana* showed extremely high susceptibility to all four insecticides evaluated: resistance ratios at the *LC*_50_ and *LC*_99_ estimates were less than 1.0 and showed that this strain was 1.5-fold to 7.4-fold more susceptible than the laboratory *P. xylostella* (S) reference (Additional file 1: Table S3). In contrast, resistance ratios at the *LC*_50_ for the field *P. xylostella* strain ranged from 2.9 for Success Neo to 41.4 for Dominex, indicating increased tolerance to all insecticides. Comparison of the *LC*_99_ estimates with commercial field doses for each insecticide implies differences in field efficacy between species. The commercial field rate of Dominex was >8-fold lower than the *LC*_99_ for *P. xylostella*, suggesting likely poor field control of this strain, but was >17-fold higher than the *LC*_99_ for *P. australiana* (Fig. 6). Control mortality was similar for the field and reference strains, averaging 3.1 to 4.4% across all bioassays.

## Discussion

Cryptic species arise when divergence does not lead to morphological change [79]. The recent discovery of a cryptic ally, *P. australiana*, to the diamondback moth, *P. xylostella*, was unexpected given the breadth of previous molecular studies of this insect. Several factors may have contributed to this discovery, including the use of light traps for specimen collection, rather than limiting sampling to *Brassica* vegetable farms. Landry and Hebert [16] also isolated DNA from legs, keeping most of each specimen intact and providing a morphological reference for examining unexpected genotypes. It is also possible that *P. australiana* was previously overlooked from nuclear DNA studies due to biases in amplification of divergent alleles. Here, we sought to determine whether *P. australiana* is an agricultural pest, and to understand its ecological and genetic differences from *P. xylostella*.

Extensive larval sampling from wild and cultivated brassicaceous plants revealed that *P. australiana* co-occurs widely with *P. xylostella* throughout southern Australia and utilizes some of the same host plants. The relative abundance of *P. australiana* was on average 9-fold lower than *P. xylostella*. We observed higher proportions of *P. australiana* in larval collections from the eastern state of New South Wales, similar to the light trap samples from Landry and Hebert [16], possibly reflecting habitat suitability. Although we did not detect *P. australiana* in limited sampling from the island state of Tasmania, the presence of brassicas in the region and evidence from light traps that wind currents can transport *Plutella* moths across Bass Strait (Lionel Hill, Pers. Comm.) suggest it is likely to occur there.

Our study confirms that the host range of *P. australiana* includes canola crops and wild brassicaceous species. In laboratory rearing, *P. australiana* completed development on sand rocket, *D. tenuifolia*, and canola, *B. napus*, and was also collected from several other wild species, though without rearing to confirm host status. Our sampling focused on relatively few introduced brassicaceous species common in agricultural areas, yet the Australian Brassicales is represented by 11 plant families [80], including several non-Brassicaceae on which *P. xylostella* and its allies have been documented feeding, such as Capparaceae [24], Cleomaceae [16] and Tropaeolaceae [23]. The Australian Brassicaceae has records for 61 genera and 205 species [80], including many introduced species but also a diversity of native genera, such as *Lepidium*, *Blennodium*, and *Arabidella*, that occur over vast areas of Australia. Wider sampling of native Brassicales may identify other suitable hosts for *P. australiana*.

*Plutella australiana* larvae were not identified among samples from sixteen commercial *Brassica* vegetable crops despite the high suitability of these crops for *P. xylostella* [81], however eggs were collected from kale. It is possible that extreme insecticide susceptibility prevents juvenile *P. australiana* populations from establishing, as commercial *Brassica* vegetable crops are typically sprayed multiple times per crop cycle [22]. Our data show that *P. australiana* is far more susceptible than *P. xylostella* to four commonly used insecticides. At commercial application rates, these insecticides are likely to provide high-level control of *P. australiana* in Australian *Brassica* crops, but some products may provide marginal or poor control against *P. xylostella* due to insecticide resistance (Fig. 6) [22, 25]. Alternatively, some vegetable cultivars may not be attractive for oviposition or suitable for larval survival in *P. australiana*. We noted that *P. australiana* cultures provided with cabbage seedlings failed to produce viable eggs over seven days, but after replacing cabbage with *Diplotaxis* seedlings, egg-laying then occurred within 24 h. Exposure to host plants stimulates reproductive behaviour in *P. xylostella* [82], but olfactory cues for host recognition or oviposition [83–85] may differ between these *Plutella* species. Host preference and performance studies are required to test these hypotheses.

Insecticide bioassays have been conducted routinely on Australian *P. xylostella* to monitor levels of insecticide resistance in field populations [22, 25]. This method appears unlikely to be affected by the presence of *P. australiana* under typical conditions, as a period of laboratory rearing is usually necessary to multiply individuals prior to screening. In our experience, laboratory rearing of the two *Plutella* species on cabbage plants selects against *P. australiana* individuals when competing with *P. xylostella* in cages, causing the complete loss of *P. australiana* within a few generations. The reasons for this are unknown but may include differences in host preference or development rate, or direct competition.

Crossing experiments revealed that hybridization can occur between *P. australiana* and *P. xylostella* under controlled conditions and is most likely to occur in crosses involving *Wolbachia*-infected *P. australiana* females. Hybridization occurs in around 10% of animal species, particularly in captivity [86], but asymmetric reproductive isolation is commonly observed in reciprocal crosses between taxa [87]. In our experiments, a strong male bias in the offspring of interspecific crosses and failure to back-cross hybrid females both follow Haldane’s rule [88], which predicts greater hybrid inviability or sterility in the heterogametic sex (female, in Lepidoptera). This pattern can arise from epistatic interactions between sex-linked and/or autosomal genes that result in genetic incompatibilities [89, 90]. Although the back-crosses with F1 hybrid females were sterile, the back-crosses with hybrid males (to both species) were viable, which could enable the transfer of genes between hybrid and/or parental species. However, it is unclear whether hybridization occurs in the wild.

Although *P. australiana* and *P. xylostella* show deep divergence (8.6%) in mtDNA [16], the sole use of mtDNA can be unreliable for inference of evolutionary history and should be corroborated using evidence from nuclear markers [34]. Our analysis revealed striking differences in nuclear diversity across the genome between co-existing populations of each *Plutella* species collected at the same locations and times, and from the same host plant species. *Plutella xylostella* populations from Australia and New Zealand have low levels of genetic diversity compared with populations from other continents, thought to reflect the recent introduction of this species from a small founding population [14, 17, 77]. Consistent with this view, we found a remarkable 1.5-fold reduction in heterozygosity across >300,000 sites in *P. xylostella* compared with sympatric *P. australiana* populations. However, both species showed limited mtDNA diversity with a single predominant haplotype. While outgroups from other continents were not available, comparative analysis of these closely-related Australian *Plutella* species suggested that patterns of mitochondrial and nuclear diversity are concordant in *P. xylostella* and consistent with a demographic bottleneck [17, 18], but discordant in *P. australiana*.

Sequence variation in mitochondrial DNA can be strongly influenced by *Wolbachia* infection [41]. Extensive *Wolbachia* screening showed that each *Plutella* species was infected with a different strain at contrasting frequencies, and fit a ‘most-or-few’ pattern whereby species infection rates are often very low (<10%) or very high (>90%) [91]. Infection incidence in *P. xylostella* was lower in Australia (1%) than previously reported across global samples (5%) [18]. Our finding of a single supergroup A strain showing 100% sequence similarity to a strain reported in *P. xylostella* from Malaysia, *plutWA1* [18], provides some support of an Asian origin for Australian *P. xylostella* [17], though does not preclude this strain also occurring elsewhere.

Fixation of infection in *P. australiana* suggests that *Wolbachia* manipulates the reproductive biology of this species. We found no evidence of sex-ratio distortion, which has been associated with a *Wolbachia* strain, *plutWB1*, in *P. xylostella* [18]. High infection can be driven by cytoplasmic incompatibility (CI) [40]. The high frequency (87%) of a single mtDNA haplotype among *P. australiana* individuals implies that the spread of *Wolbachia* infection has driven a selective sweep of co-inherited mtDNA through the population, causing a loss of mtDNA diversity [41]. High nuclear diversity (relative to sympatric *P. xylostella*) supports this hypothesis, because a demographic bottleneck should reduce diversity across the entire genome [34].

*Plutella australiana* and *P. xylostella* have co-existed in Australia for at least 125 years ($\geqslant $1300 generations), yet have strongly divergent mitochondrial and nuclear genomes, *Wolbachia* infections and insecticide susceptibility phenotypes. Our observations during laboratory rearing and crossing experiments also suggested that interspecific differences in host plant use may exist. What explains such strong divergence between the two *Plutella* species, given sympatry and the capacity to hybridize? Endemism of *P. australiana* [16] implies an ancient evolutionary history in Australia, and our data provide support for existing views that Australian *P. xylostella* were recently introduced from a small ancestral source population, possibly from Asia [17, 18, 77]. Therefore, the two *Plutella* species may have diverged in allopatry and recently come into secondary contact. Maintenance of divergence suggests strong continuing reproductive isolation, which can evolve as a side-effect of allopatric divergence [44]. All 99 individuals that were RAD sequenced showed concordance in nuclear multilocus genotypes and mtDNA genotypes identified through PCR-RFLP regardless of geographic location, as shown by STRUCTURE analysis. Cryptic species in sympatry provides strong evidence of limited genetic exchange [79]. A small degree of genotypic admixture evident for a few individuals in the STRUCTURE plots might be explained by ancestral polymorphism or introgressive hybridization [28], or alternatively, could be an artefact if our dataset is not representative of the entire genetic background [33]. The level of hybridization that may be occurring between these species is unknown. Isolation may not be uniform across the genome [92, 93], and scans of larger genomic regions may be required to identify introgression and detect hybrids.

The factors leading to reproductive isolation between the two *Plutella* species in nature are unknown but could include a range of pre- or post-mating isolation mechanisms, such as assortive mating or hybrid fitness costs. Behavioural mating choices are often the main isolating factor in sympatric animals [86]. Does *Wolbachia* cause a reproductive barrier? The contrast in infection status creates the potential for cytoplasmic incompatibility between species [94]. Interspecific crosses showed a pattern of asymmetric isolation consistent with the expected effects of unidirectional CI, where 21% crosses involving infected *P. australiana* females produced viable offspring, while the reciprocal CI-cross direction (uninfected *P. xylostella* females crossed with infected *P. australiana* males) was nearly sterile. However, this pattern was not continued in the F1 generation: infected hybrid males (derived from the *P. australiana* maternal line) produced offspring at comparable rates when back-crossed to either uninfected *P. xylostella* or infected *P. australiana* female parents. The role of *Wolbachia*-induced postzygotic isolation between the two *Plutella* species requires further study, though our results suggest it could be more important in the F0 generation. *Wolbachia* can contribute to post-zygotic genetic isolation after speciation by complementing hybrid incompatibilities [94, 95]. Symbiont infections could also influence mating behaviour and contribute to pre-mating isolation [96].

## Conclusions

The discovery of cryptic pest species introduces complexities for their management and also exciting opportunities for understanding ecological traits. We found strong genomic and phenotypic divergence in two cryptic mitochondrial *Plutella* lineages co-existing in nature, supporting their status as distinct species [16] despite the capacity to hybridize. Reproductive isolation is likely to have evolved during allopatric speciation, and genome-wide sequence data suggest it has been maintained following secondary contact. Variation in *Wolbachia* infections might be one factor reinforcing reproductive barriers.

*Plutella australiana* co-occurs with *P. xylostella* throughout agricultural regions of southern Australia, but made up only 10% of *Plutella* juveniles collected from cultivated and wild brassicaceous plants. A lack of population structure across neutral SNP markers suggests that *P. australiana* populations are linked by high levels of gene flow, and also that *P. australiana* is a highly mobile species, which is supported by light trap collections [16] and seasonal colonization of canola crops. Future molecular analysis of Australian *Plutella* should include a species identification step using a molecular diagnostic assay. For ecological studies, it may be possible to perform molecular species identification to confidently distinguish a representative sub-sample of individuals or pooled samples. Our study has shown that while *P. australiana* can attack canola crops, there is no evidence of pest status in commercial *Brassica* vegetables crops, and bioassays suggested that field populations should be easily controlled with insecticides. Though *P. australiana* is a potential pest of some Australian *Brassica* crops, it is of secondary importance to the diamondback moth, *P. xylostella*.

## Additional file

Additional file 1 **Table S1** The four variable nucleotide sites among the five *P. xylostella* 613 bp COI haplotypes identified in 102 individuals from Australia. **Table S2** The eight variable nucleotide sites among the nine *P. australiana* 613 bp COI haplotypes identified in 87 individuals from Australia. **Table S3** Log-logistic regression statistics for dose-response bioassays on *P. australiana* and *P. xylostella* field strains and the *P. xylostella* reference strain exposed to four commercial insecticides. (PDF 91 kb)
