# Supplementary material for: Cryptic Plutella species show deep divergence despite the capacity to hybridize
Source: BMC Evol Biol. 2018 May 29;18:77. doi: 10.1186/s12862-018-1183-4 (PMC5975261; doi:10.1186/s12862-018-1183-4)
Supplement: Supplementary file 1 — Table S1 The four variable nucleotide sites among the five P. xylostella 613 bp COI haplotypes identified in 102 individuals from Australia. Table S2 The eight variable nucleotide sites among the nine P. australiana 613 bp COI haplotypes identified in 87 individuals from Australia. Table S3 Log-logistic regression statistics for dose-response bioassays on P. australiana and P. xylostella field strains and the P. xylostella reference strain exposed to four commercial insecticides. (PDF 91 kb) [file 12862_2018_1183_MOESM1_ESM.pdf]

Additional File 1

Table S1: The four variable nucleotide sites among the five *P. xylostella* 613 bp COI haplotypes identified in 102 individuals from Australia. Shown are sequences from this study and re-analysed sequences from Landry and Hebert [1] downloaded from dx.doi.org//10.5883//DS-PLUT1. Three haplotypes correspond to those reported by Saw et al. [2]: PxCOI01/PxMt01, GenBank accession: DQ394347; PxCOI02/PxMt06, GenBank accession: DQ394352; PxCOI04/PxMt02, GenBank accession: DQ394348. Nucleotide positions were determined from sequence MF151841. Only positions that differ from haplotype PxCOI01 are shown.

	Nucleotide position					
Haplotype	343	373	541	598	No. individuals	Sequence reference
PxCOI01	T	A	A	A	76	MF151841
PxCOI02	C	.	.	.	1	PHSAU1068-12
PxCOI03	.	G	.	.	1	LOQTC311-07
PxCOI04	.	.	G	.	23	MF151838
PxCOI05	.	.	.	G	1	PHLCD068-12

Table S2: The eight variable nucleotide sites among the nine *P. australiana* 613 bp COI haplotypes identified in 87 individuals from Australia. Haplotypes PaCOI01 and PaCOI02 were identified among sequences from this study and Landry and Hebert [1], and PaCOI04, PaCOI05, PaCOI08 and PaCOI09 were identified from Landry and Hebert [1]. Nucleotide positions were determined from sequence MF151865. Only the positions that differ from haplotype PaCOI01 are shown.

	Nucleotide position									
Haplotype	202	278	316	382	400	433	481	519	No. individuals	Sequence reference
PaCOI01	T	T	A	C	C	A	G	G	74	MF151865
PaCOI02	C	.	.	.	.	.	.	.	6	MF151831
PaCOI03	C	.	.	.	.	.	A	.	1	MF151885
PaCOI04	C	.	.	.	.	.	.	A	1	MCCAA2949-12
PaCOI05	.	C	.	.	.	.	.	.	1	PHLCA920-11
PaCOI06	.	.	G	.	.	.	.	.	1	MF151836
PaCOI07	.	.	.	A	.	.	.	.	1	MF151883
PaCOI08	.	.	.	.	T	.	.	.	1	LSM1299-11
PaCOI09	.	.	.	.	.	G	.	.	1	LNSWA731-05

Table S3: Log-logistic regression statistics for dose-response bioassays on *P. australiana* (*P. aus*) and *P. xylostella* (*P. x*) field strains and the *P. xylostella* (S) laboratory reference strain exposed to four commercial insecticides.

Product	Strain	n	Slope (SE)	LC_{50} (95% CL) [$\mathrm{mg\,L^{-1}}$ a.i.]	RR_{LC50}	LC_{99} (95% CL) [$\mathrm{mg\,L^{-1}}$ a.i.]	RR_{LC99}
Coragen	*P. aus*	320	2.016 ± 0.236	0.028 (0.023–0.034)	0.45	0.276 (0.161–0.474)	0.22
	P. x	322	1.363 ± 0.149	0.524 (0.411–0.667)	8.26	15.235 (7.374–31.479)	11.88
	P. x (S)	323	1.528 ± 0.165	0.063 (0.051–0.079)	1.00	1.282 (0.666–2.47)	1.00
Dominex	*P. aus*	320	1.078 ± 0.117	0.032 (0.024–0.042)	0.13	2.267 (0.92–5.583)	0.16
	P. x	320	1.292 ± 0.146	9.792 (7.563–12.679)	41.38	343.317 (158.25–744.816)	24.85
	P. x (S)	320	1.130 ± 0.118	0.237 (0.182–0.308)	1.00	13.815 (5.685–33.574)	1.00
Proclaim	*P. aus*	320	2.073 ± 0.235	0.012 (0.01–0.015)	0.68	0.111 (0.066–0.186)	0.39
	P. x	320	1.254 ± 0.146	0.073 (0.056–0.096)	4.15	2.868 (1.282–6.415)	10.04
	P. x (S)	320	1.652 ± 0.181	0.018 (0.014–0.022)	1.00	0.286 (0.153–0.532)	1.00
Success Neo	*P. aus*	320	2.087 ± 0.293	0.011 (0.009–0.014)	0.14	0.101 (0.056–0.184)	0.14
	P. x	320	1.766 ± 0.196	0.242 (0.197–0.297)	2.94	3.266 (1.805–5.912)	4.65
	P. x (S)	321	2.143 ± 0.255	0.082 (0.068–0.099)	1.00	0.703 (0.417–1.184)	1.00

n, number of insects tested; LC_{50}, dose predicted to cause 50% mortality with 95% confidence limits; LC_{99}, dose predicted to cause 99% mortality with 95% confidence limits; RR_{LC50}, resistance ratio at the LC_{50} dose level; RR_{LC99}, resistance ratio at the LC_{99} dose level.

References

[1] Landry JF, Hebert PDN. *Plutella australiana* (Lepidoptera, Plutellidae), an overlooked diamondback moth revealed by DNA barcodes. ZooKeys. 2013;(327):43–63.

[2] Saw J, Endersby NM, McKechnie SW. Low mtDNA diversity among widespread Australian diamondback moth *Plutella xylostella* (L.) suggests isolation and a founder effect. Insect Sci. 2006;13(5):365–373.
